# The Platelet Activating Factor–Platelet Activating Factor Acetylhydrolase Enzyme Axis in Anaphylaxis: Current Evidence and Future Perspectives

**DOI:** 10.3390/ijms27177783

**Published:** 2026-08-31

**Authors:** Arianna Vignini, Sabrina Costanzo, Matteo Martini, Sonila Alia, Valentina Membrino, Tiziana Di Crescenzo, Elena Buti, Maria Beatrice Bilò

**Affiliations:** 1 Department of Clinical Sciences, Università Politecnica delle Marche, 60126 Ancona, Italy; s.alia@staff.univpm.it (S.A.); v.membrino@pm.univpm.it (V.M.); t.dicrescenzo@pm.univpm.it (T.D.C.); 2 Postgraduate School of Allergy and Clinical Immunology, Marche Polytechnic University, 60126 Ancona, Italy; sabrina.costanzo098@gmail.com (S.C.); elena.buti@ospedaliriuniti.marche.it (E.B.); m.b.bilo@staff.univpm.it (M.B.B.); 3 Allergy Unit, Department of Internal Medicine, University Hospital AOU delle Marche, 60126 Ancona, Italy; matteo.martini@ospedaliriuniti.marche.it; 4 Department of Clinical and Molecular Sciences, Marche Polytechnic University, 60126 Ancona, Italy

**Keywords:** anaphylaxis, platelet-activating factor (PAF), platelet-activating factor acetylhydrolase (PAF-AH), hymenoptera venom allergy, biomarkers, therapeutic targets

## Abstract

Anaphylaxis is a severe systemic hypersensitivity reaction characterized by rapid onset, unpredictable clinical course, and potentially fatal outcomes. Although its diagnosis remains primarily clinical, the identification of biomarkers capable of improving risk stratification and understanding disease mechanisms remains an important unmet need. Among the mediators implicated in anaphylaxis, platelet-activating factor (PAF) has emerged as a key effector molecule involved in vascular permeability, bronchoconstriction, platelet activation, and cardiovascular dysfunction. The biological activity of PAF is tightly regulated by platelet-activating factor acetylhydrolase (PAF-AH), the enzyme responsible for its degradation, making the PAF–PAF-AH axis a potentially important determinant of reaction severity. OBJECTIVES: The present review summarizes current knowledge regarding the biology of PAF and PAF-AH and examines their role in the pathophysiology of anaphylaxis, with particular emphasis on Hymenoptera venom allergy (HVA). The therapeutic potential of targeting the PAF pathway is also discussed. Experimental and clinical evidence consistently suggests an association between increased PAF activity, reduced PAF-AH activity, and more severe anaphylactic reactions. However, methodological limitations, biological variability, and conflicting findings across studies currently limit the clinical applicability of these molecules as standalone biomarkers. Overall, the PAF–PAF-AH axis represents a biologically plausible link between molecular mechanisms and clinical severity in anaphylaxis. Further studies are required to standardize measurements, validate clinical utility, and define the role of this pathway as both a biomarker and therapeutic target; hence gaining insight into PAF’s role in various biological processes is critical.

## 1. Introduction

Anaphylaxis is a severe, potentially life-threatening systemic hypersensitivity reaction characterized by its rapid onset and unpredictable clinical course. Despite more than a century of investigation since the term was first introduced by Portier and Richet in 1902, anaphylaxis continues to represent a clinical condition that is challenging to predict, diagnose, and manage effectively. Current international guidelines define anaphylaxis as a serious systemic reaction that is usually rapid in onset and may cause death, resulting from either immunologic mechanisms, including immunoglobulin E (IgE)-mediated and non-IgE-mediated pathways, or the direct activation of effector cells through non-immunologic mechanisms [1,2]. The epidemiology of anaphylaxis has changed substantially over recent decades. Several population-based studies have demonstrated a continuous increase in the incidence of anaphylaxis worldwide, particularly in industrialized countries. Although mortality remains relatively low, estimated at approximately 0.5–1 deaths per million persons annually [3], the burden on healthcare systems is considerable due to increasing emergency department visits and hospitalizations [4,5]. Moreover, the fear of recurrent episodes significantly impairs quality of life and represents an important psychosocial burden for affected individuals and their families [6]. Due to its unpredictable nature and possible fatal outcome, anaphylaxis—and even the risk of its occurrence—constitutes a significant source of concern for both patients and healthcare professionals. Several risk factors for anaphylaxis have been identified, like age, asthma, mastocytosis, elevated baseline serum tryptase concentrations, and genetic predispositions involving mast cell activation pathways [7,8]. However, predicting which individuals will experience severe anaphylaxis remains difficult, highlighting the need for reliable biomarkers capable of stratifying risk and guiding management. The number of potential anaphylaxis triggers is extensive, but the most common are food allergens, followed by medication and the venom from a stinging insect (order Hymenoptera) [9]. Hymenoptera venom allergy (HVA), caused by stings from bees, wasps, hornets, represents one of the leading and stable causes of severe anaphylaxis in adults in Europe and is associated with a substantial risk of recurrent systemic reactions [9,10]. HVA is an often underestimated condition and a significant cause of morbidity and mortality worldwide. Epidemiological studies indicate that systemic reactions occur in approximately 3–8% of adults following Hymenoptera stings, while fatal reactions, although uncommon, continue to be reported worldwide. Clinical manifestations range from mild local reactions to severe to potentially fatal anaphylaxis [10,11]. Importantly, venom-induced anaphylaxis often occurs in otherwise healthy individuals and may constitute the first manifestation of an underlying clonal mast cell disorder.

Regardless of the underlying mechanism, an anaphylactic reaction implies the release of mediators with vasoactive, pro-inflammatory, and chemotactic activities. Preformed and newly formed biochemical molecules have been identified as mediators of anaphylaxis. In classical IgE-mediated reactions, allergen cross-linking of FcεRI-bound IgE antibodies induces rapid degranulation and the release of preformed mediators including histamine, tryptase, chymase, heparin, and carboxypeptidase A3 [12]. The literature on anaphylaxis also highlights other biochemical molecules that can play an important role in the pathogenesis of this condition: newly synthesized mediators, including prostaglandins, leukotrienes, thromboxanes, platelet-activating factor (PAF), and various cytokines and chemokines, which further amplify the allergic and inflammatory response [12]. Although histamine has historically been considered the principal mediator of anaphylaxis, particular emphasis is placed on the roles of PAF, an endogenous phospholipid involved in inflammation, immune responses, and platelet aggregation [13].

PAF is a potent phospholipid mediator synthesized by a variety of cells, including mast cells, basophils, eosinophils, neutrophils, monocytes, macrophages, endothelial cells, and platelets. Through binding to a specific G-protein-coupled receptor (PAFR), PAF induces vasodilation, increased vascular permeability, bronchoconstriction, leukocyte recruitment, platelet aggregation, and hypotension—hallmark features of severe anaphylaxis [13,14]. Experimental studies have demonstrated that PAF is substantially more potent than histamine in inducing vascular leakage and shock, suggesting a central role in life-threatening manifestations of anaphylaxis.

**Figure 1:**
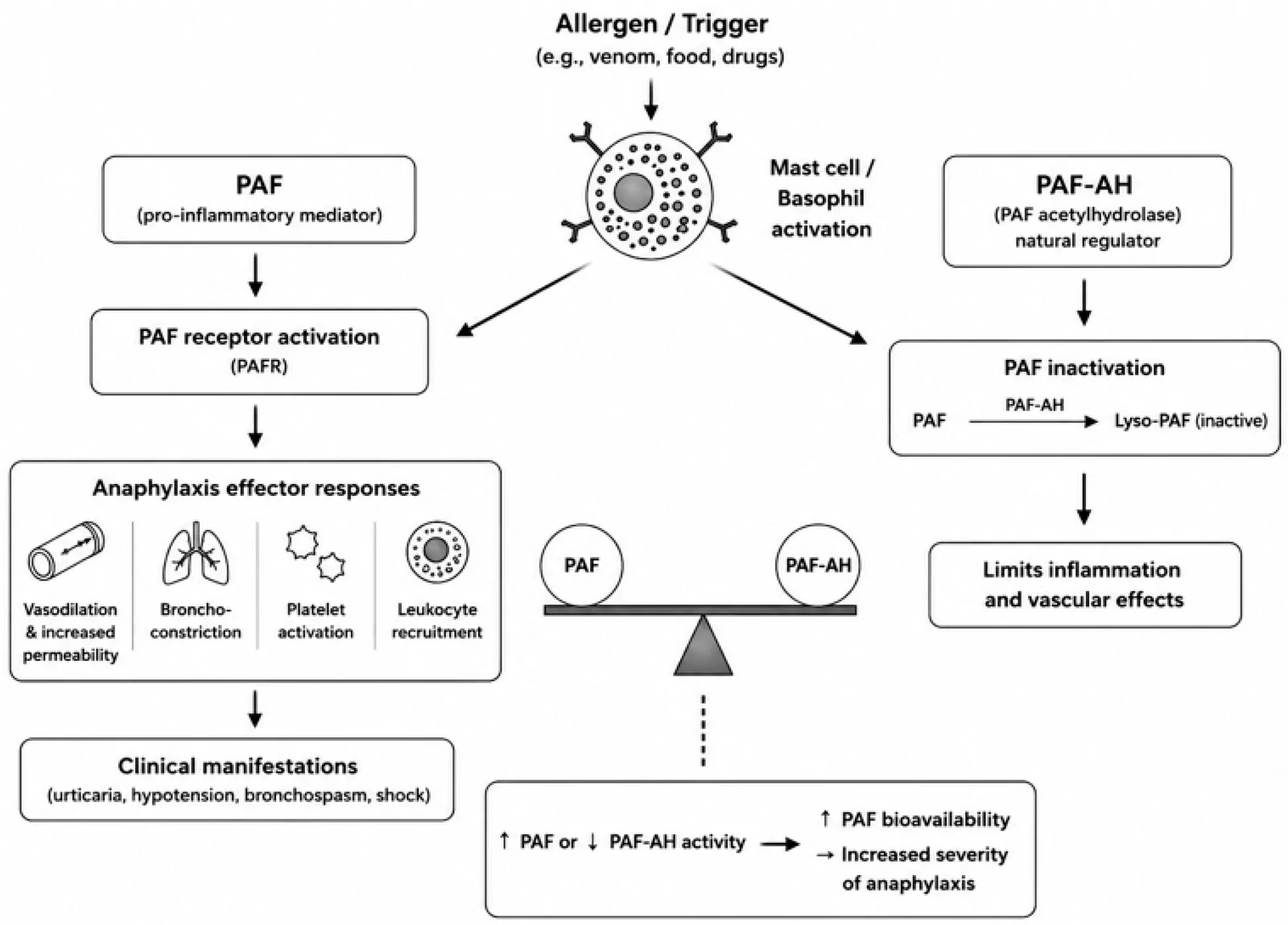
Balance between PAF and PAF-AH in the regulation of anaphylaxis. PAF generated following mast cell and basophil activation promotes key effector mechanisms of anaphylaxis, including vascular leakage, bronchoconstriction, platelet activation, and leukocyte recruitment. PAF-AH counteracts these effects by degrading PAF into inactive lyso-PAF. The relative balance between PAF bioavailability and PAF-AH activity contributes to the severity of anaphylactic reactions.

The biological activity of PAF is tightly regulated by platelet-activating factor acetylhydrolase (PAF-AH), also known as lipoprotein-associated phospholipase A2 (Lp-PLA2), a circulating enzyme responsible for hydrolyzing and inactivating PAF. By degrading the acetyl group at the sn-2 position, PAF-AH terminates PAF signaling and contributes to maintaining immune homeostasis. By limiting PAF bioavailability, PAF-AH contributes to the regulation of inflammatory and vascular responses. Consequently, the PAF–PAF-AH axis has attracted increasing interest as a potential determinant of anaphylaxis severity and as a source of novel biomarkers and therapeutic targets (Figure 1).

Despite growing evidence supporting the importance of PAF in anaphylaxis, significant knowledge gaps remain. The mechanisms regulating PAF synthesis and degradation during allergic reactions are incompletely understood, standardized methods for measuring circulating PAF and PAF-AH are lacking, and the clinical applicability of these biomarkers has yet to be fully established. Furthermore, emerging experimental data suggest that pharmacological modulation of the PAF signaling pathway may represent a promising therapeutic strategy for severe allergic diseases and anaphylaxis [15]. Given these considerations, a comprehensive understanding of the biology of PAF and PAF-AH is increasingly important.

Hence, gaining insight into PAF’s role in various biological processes is critical. We conducted a comprehensive narrative review of the literature to provide an overview of current knowledge on PAF and PAF-AH biology, with particular emphasis on their involvement in anaphylaxis and their potential roles as biomarkers of clinical severity and therapeutic targets.

## 2. Biology of the PAF–PAF-AH Axis

PAF, chemically known as 1-O-alkyl-2-acetyl-sn-glycero-3-phosphocholine (AGEPC or PAF-acether), is a highly active phospholipid mediator that plays a key role in the pathogenesis of many immune and inflammatory disorders [16]. It was first introduced into the scientific literature in 1966, when Barbaro and Zvaifler reported a substance capable of triggering antigen-induced histamine release from rabbit platelets producing antibodies in passive cutaneous anaphylaxis [17]. Nearly four years later, Henson described a “soluble factor” released by leukocytes that stimulated the liberation of vasoactive amines from platelets [18]. Subsequent studies demonstrated that PAF receptors (PAFRs) are expressed on a wide range of cell types, particularly those involved in innate and adaptive immunity, such as basophils, mast cells, macrophages, and monocytes, as well as neutrophils, eosinophils, platelets, and endothelial cells [19]. Although a de novo synthetic pathway exists, in most cells, biologically active PAF predominantly arises from membrane phospholipid remodeling. In this pathway, phospholipase A_2_ (PLA_2_) acts primarily on phosphatidylcholine (PC), leading to the release of arachidonic acid (AA) and the formation of lysophosphatidylcholine (LPC). The newly generated LPC is subsequently acetylated by LPC acetyltransferase (LPCAT) to form PAF. This pathway enables rapid PAF generation in response to inflammatory stimuli, making it particularly relevant during acute hypersensitivity reactions. Functionally, PAF mediates its effects via engagement of its specific G-protein-coupled receptor, PAFR, which is expressed on numerous immune and non-immune cells.

Receptor activation initiates intracellular signaling events that culminate in Ca^2+^-dependent activation of protein kinase C (PKC), ultimately leading to the amplification of inflammatory responses. Since the majority of PAF-producing cells also express PAFR, these findings support a role for autocrine and paracrine signaling in mediating many of PAF’s biological functions [20].

Activation of PAFR initiates intracellular signaling pathways that regulate vascular tone, endothelial permeability, leukocyte recruitment, platelet activation, and smooth muscle contraction. These biological activities place PAF at the interface between inflammation, immunity, and vascular homeostasis, highlighting its potential relevance in a variety of inflammatory and hypersensitivity disorders.

Given its powerful biological activity, the biological activity of PAF is tightly controlled by PAF-AH, present in both plasma and cytosolic compartments, a family of Ca^2+^-independent phospholipase A_2_ enzymes.

At least three distinct isoforms of PAF-AH have been identified: one extracellular, known as the plasma-type, and two intracellular, referred to as tissue-type enzymes. The plasma isoform, a 45-kDa monomeric enzyme that circulates primarily bound to plasma lipoproteins, represents the principal mechanism for systemic PAF degradation [21]. Among the intracellular forms, isoform Ib is a heterotrimeric enzyme composed of three subunits—α (45 kDa), β (30 kDa), and γ (29 kDa) [22]. In contrast, isoform II is a 40-kDa monomeric protein that shares approximately 41% amino acid sequence identity with the plasma-type enzyme [23].

By hydrolyzing the acetyl group at the sn-2 position of PAF to its inactive metabolite, lyso-PAF, PAF-AH serves as a critical regulatory mechanism that limits the duration and magnitude of PAF-mediated signaling, thereby modulating its effects on inflammation, platelet activation, and vascular responses. PAF has a remarkably short elimination half-life, ranging from 3 to 13 min and is quickly degraded by PAF-AH [24].

Hence, circulating PAF concentrations are largely regulated by PAF-AH activity, which plays a key role in determining the molecule’s short half-life and serves as a critical endogenous regulator limiting both the magnitude and duration of PAF-mediated responses. By controlling PAF bioavailability, PAF-AH contributes to the maintenance of vascular homeostasis and prevents excessive inflammatory activation [25,26].

Consequently, the PAF–PAF-AH axis has attracted increasing interest not only as a mechanistic pathway underlying anaphylaxis, but also as a potential source of biomarkers for risk stratification and novel therapeutic targets [12].

**Figure 2:**
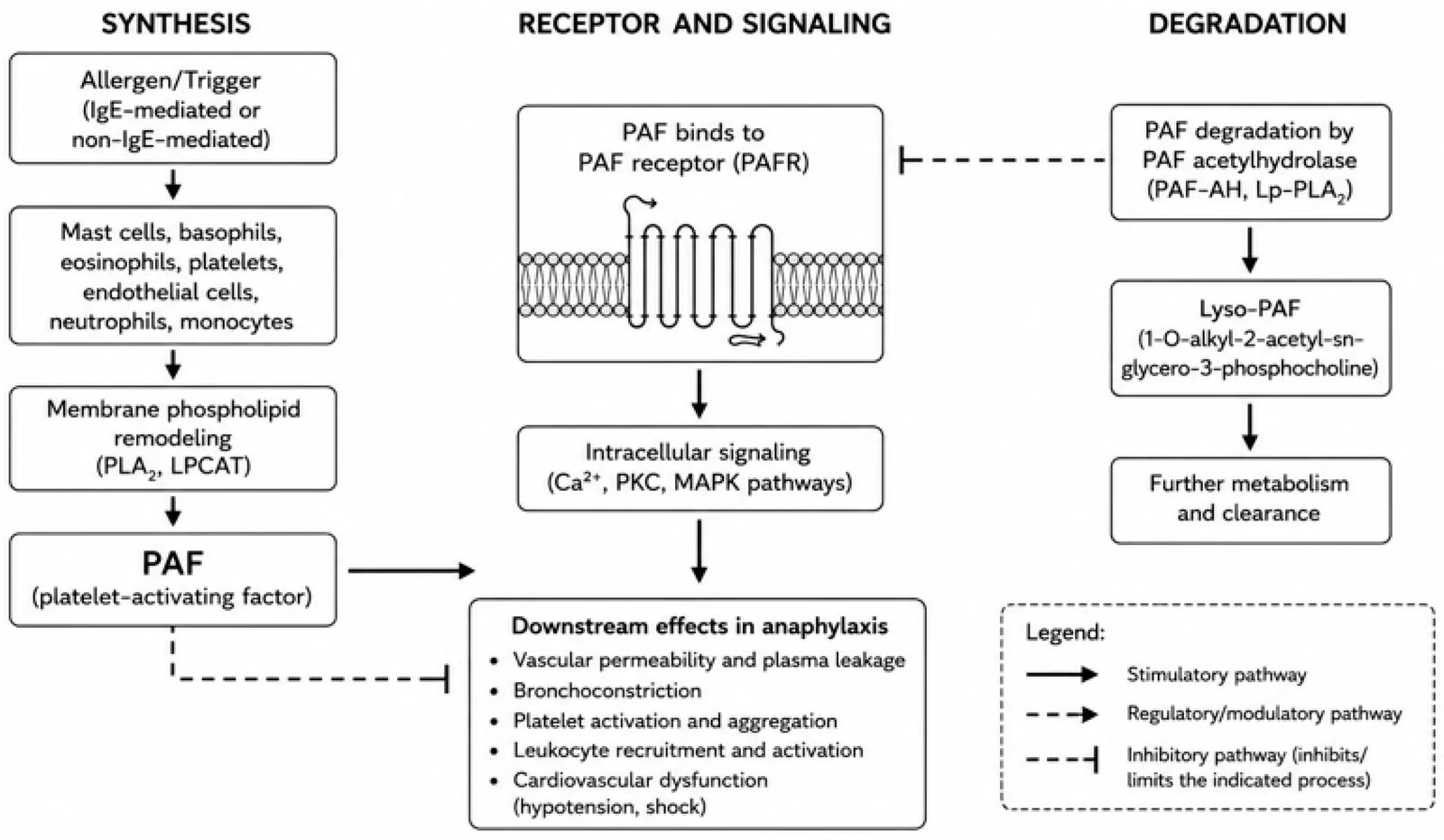
The PAF–PAF-AH axis in anaphylaxis. Schematic representation of the biological pathway of platelet-activating factor (PAF), from its synthesis in activated cells to receptor-mediated signaling and downstream clinical effects, and its degradation by PAF acetylhydrolase (PAF-AH, Lp-PLA_2_) to lyso-PAF and further metabolites, leading to the termination of PAF activity and resolution of the inflammatory response.

Taken together, the tight regulation of PAF bioavailability by PAF-AH highlights the importance of the PAF–PAF-AH axis in maintaining immune and vascular homeostasis. Alterations in this balance may have important pathophysiological consequences and have therefore become the subject of increasing investigation in allergic diseases and anaphylaxis (Figure 2).

## 3. Specific Role of PAF in Anaphylaxis

PAF is now recognized as a major effector mediator of anaphylaxis and has been extensively investigated in both experimental and clinical settings [14,19,27].

PAF receptor activation triggers intracellular calcium mobilization and multiple signaling pathways, including protein kinase C and mitogen-activated protein kinases, leading to the production of arachidonic acid metabolites and other inflammatory mediators that amplify the response [28,29]. Beyond its direct vascular and inflammatory effects, PAF can further amplify immune and inflammatory responses, including allergic reactions, through interactions with mast cells. Kajiwara et al. demonstrated that PAF induces histamine release from human lung mast cells through PAFR activation, providing evidence of a positive feedback loop capable of enhancing mediator release during anaphylaxis [30]. These findings further support the concept that PAF functions not only as an effector mediator, but also as an amplifier of mast cell-driven allergic inflammation.

The biological actions of PAF closely mirror the clinical manifestations of anaphylaxis. Through its effects on vascular permeability, smooth muscle contraction, platelet activation, and leukocyte recruitment, PAF contributes to hypotension, bronchospasm, tissue edema, and cardiovascular collapse. Importantly, accumulating evidence suggests that circulating PAF levels correlate more closely with reaction severity than classical mediators such as histamine or serum tryptase [31].

Experimental studies have provided compelling evidence for a causal role of PAF in anaphylactic shock. In murine models, the administration of recombinant human plasma-type PAF-AH significantly reduced hypotension and mortality during systemic anaphylaxis, highlighting the importance of PAF bioavailability in determining clinical outcomes [32].

More recently, Suzuki et al., in a murine model, demonstrated that LPLAT9-dependent PAF synthesis in mast cells contributes to IgE-mediated cutaneous anaphylaxis, further strengthening the mechanistic link between PAF signaling and allergic disease [33].

The strongest evidence supporting a role for the PAF–PAF-AH axis in human anaphylaxis comes from clinical studies. In a landmark investigation, Vadas et al. demonstrated that circulating PAF levels increase in parallel with anaphylaxis severity, whereas PAF-AH activity shows a significant inverse correlation. Patients with severe or fatal reactions exhibited the highest PAF concentrations and the lowest PAF-AH activity, suggesting that impaired degradation of PAF may contribute directly to severe clinical manifestations [34].

Subsequent studies have largely confirmed these observations. Brown et al. reported that reduced PAF-AH activity was associated with more severe reactions characterized by hypotension and hypoxemia, supporting the hypothesis that the host’s capacity to degrade PAF influences the severity of anaphylaxis [35].

Similarly, Upton et al. demonstrated that low PAF-AH activity is associated with life-threatening pediatric anaphylaxis and intensive care admission, highlighting its potential utility as a biomarker for risk stratification [36]. Particular interest has focused on Hymenoptera venom allergy, in which PAF-AH has been investigated as a potential predictor of severe systemic reactions. Pravettoni et al. first demonstrated an inverse relationship between baseline PAF-AH activity and anaphylaxis severity in patients with Hymenoptera venom allergy, suggesting that reduced enzymatic activity may represent a predisposing factor for severe venom-induced reactions [37]. Subsequent studies have generally supported an association between reduced PAF-AH activity and increased susceptibility to anaphylaxis, although not all investigations have found a consistent relationship between enzyme activity and reaction severity, indicating that additional immunological and clinical factors contribute to the complexity of anaphylactic responses [38,39] (Table 1).

Taken together, the available experimental and clinical evidence supports a biologically relevant association between the PAF–PAF-AH axis and anaphylaxis severity. Across different clinical settings, increased PAF concentrations and/or reduced PAF-AH activity have generally been associated with more severe reactions, particularly those characterized by hypotension, hypoxemia, or cardiovascular compromise [34,35,36,37,38]. Nevertheless, the evidence is not entirely consistent. Differences in study populations, anaphylaxis triggers, timing of sample collection, severity grading systems, and analytical methods for PAF or PAF-AH assessment limit direct comparisons across studies [27,38,39]. This heterogeneity is particularly evident in Hymenoptera venom allergy, in which reduced PAF-AH activity has been associated with severe reactions in some cohorts but not in others [37,38,39]. Therefore, although the PAF–PAF-AH axis represents a compelling mechanistic and biomarker candidate, prospective multicenter studies using standardized assays and harmonized clinical endpoints are required before its prognostic value can be established [27,40,41].

## 4. Limitations of PAF and PAF-AH as Biomarkers of Anaphylaxis

Despite the growing body of evidence supporting a role for the PAF–PAF-AH axis in anaphylaxis, several limitations currently hinder the clinical implementation of these molecules as reliable biomarkers. Although numerous studies have reported an association between elevated PAF levels, reduced PAF-AH activity, and increased reaction severity, the findings have not been entirely consistent across different patient populations and clinical settings [27,28,36,40].

Moreover, a recent systematic review and meta-analysis evaluating the diagnostic utility of biomarkers in anaphylaxis highlighted the limited amount of available evidence for both PAF and PAF-AH, emphasizing the need for further studies before their diagnostic role can be established [41].

One of the major challenges relates to the measurement of circulating PAF itself. PAF is characterized by an extremely short half-life, rapid metabolism, and low circulating concentrations, making accurate quantification technically demanding [24]. Furthermore, PAF levels may be significantly influenced by the timing of sample collection relative to symptom onset, introducing substantial variability among studies. As a result, direct PAF measurement is currently restricted to specialized research laboratories and is not feasible for routine clinical use [40].

Measurement of PAF-AH activity offers several practical advantages over direct PAF quantification because the enzyme is more stable in circulation and can be assessed even after resolution of the acute event. Nevertheless, important methodological issues remain unresolved. Different studies have employed distinct analytical techniques, sample processing protocols, and reference ranges, making the direct comparison of results difficult. The lack of standardized assays and universally accepted cut-off values represents a major obstacle to clinical implementation [28,42].

Additional complexity arises from the biological characteristics of PAF-AH itself. Plasma PAF-AH, also known as lipoprotein-associated phospholipase A_2_ (Lp-PLA_2_), circulates predominantly bound to low-density and high-density lipoproteins. Consequently, enzyme activity may be influenced by lipid metabolism, cardiovascular risk factors, inflammatory conditions, and concomitant diseases and medications, common in real-world populations, potentially confounding its interpretation as a specific marker of anaphylaxis severity [29,43].

Another important limitation is the heterogeneity of anaphylaxis itself, which is actually an umbrella definition for several different endotypes [44,45]. Clinical severity is determined by the interaction of multiple mediators, including histamine, tryptase, leukotrienes, prostaglandins, cytokines, complement-derived factors, and platelet-derived mediators, in addition to patient-related factors such as age, comorbidities, underlying mast cell disorders, and genetic susceptibility [12,40]. Therefore, it is unlikely that a single biomarker can fully capture the biological complexity underlying severe reactions caused by different pathogenetic pathways. This concept is supported by studies in Hymenoptera venom allergy, where reduced baseline PAF-AH activity has been associated with severe systemic reactions in some cohorts but not consistently across all investigations [37,38,39].

Such discrepancies likely reflect differences in study design, patient selection, severity classification systems, and laboratory methodologies. Given these limitations, PAF and PAF-AH should not be considered in isolation but rather within the broader context of currently available and emerging biomarkers of anaphylaxis. Although several mediators have been investigated, each presents specific advantages and limitations with respect to diagnostic utility, prognostic value, analytical feasibility, and clinical applicability. A comparison of the principal biomarkers currently studied in anaphylaxis is summarized in Table 2.

Although the direct measurement of circulating PAF would theoretically provide the most direct assessment of pathway activation, its short half-life and technical challenges limit its clinical applicability. Consequently, PAF-AH has attracted greater interest as a biomarker candidate because its activity can be measured more reliably and remains relatively stable after resolution of the acute event.

## 5. Therapeutic Targeting of the PAF Pathway

The strong mechanistic association between PAF signaling and anaphylaxis severity has stimulated considerable interest in the development of therapeutic strategies aimed at modulating this pathway. Unlike traditional approaches that primarily target histamine-mediated effects, interventions directed against PAF may potentially address multiple pathophysiological mechanisms involved in severe anaphylaxis, including vascular leakage, bronchoconstriction, platelet activation, and cardiovascular collapse [19,40].

Several classes of PAFR antagonists have been developed and evaluated in experimental models of allergic and inflammatory diseases. Early studies demonstrated that pharmacological blockade of PAF signaling reduced airway hyperresponsiveness, inflammatory cell recruitment, and tissue injury in various experimental settings [26,46]. However, despite encouraging preclinical results, the clinical development of many PAF antagonists has been limited by suboptimal efficacy, pharmacokinetic challenges, and difficulties in translating experimental findings into human disease [14]. Particularly compelling evidence has emerged from animal models of anaphylaxis. In a murine model of peanut-induced anaphylaxis, concurrent inhibition of both histamine and PAF pathways provided significantly greater protection against severe reactions than blockade of either pathway alone, suggesting that these mediators act synergistically during anaphylactic responses [47]. These findings support the concept that targeting multiple mediators simultaneously may represent a more effective therapeutic strategy than focusing on a single pathway.

An alternative approach involves enhancing the degradation of endogenous PAF. Experimental administration of recombinant plasma-type PAF-AH has been shown to reduce hypotension and mortality in animal models of anaphylactic shock, providing proof-of-concept that the modulation of PAF metabolism can influence clinical results [32]. Although these observations have not yet resulted in approved therapies for anaphylaxis, they highlight the therapeutic potential of manipulating the PAF–PAF-AH axis.

Recent advances in lipidomics and molecular immunology have renewed interest in the development of more selective PAF-targeted interventions. Improved understanding of PAF biosynthesis, receptor signaling, and enzymatic degradation may facilitate the identification of novel pharmacological targets capable of selectively modulating PAF activity while minimizing off-target effects [20,33]. Despite these promising developments, significant challenges remain. The redundancy of inflammatory pathways involved in anaphylaxis, the rapid onset of clinical symptoms, the different endotypes of anaphylaxis, and the complexity of mediator networks may limit the effectiveness of therapies directed exclusively at PAF signaling. Consequently, PAF-targeted interventions are more likely to emerge as adjunctive rather than replacement therapies for current management strategies [14,40].

Beyond the development of PAF-targeted therapies, the PAF–PAF-AH axis may also have potential implications for personalized medicine. Characterization of this axis may eventually contribute to the identification of patients with a biological predisposition to severe anaphylaxis [12,34,36,37]. Rather than being considered as a standalone biomarker, PAF-AH activity could potentially be integrated with clinical risk factors and other established or emerging biomarkers to generate multimarker risk profiles reflecting different anaphylaxis phenotypes and endotypes [12,27,42]. Such an approach may be particularly relevant in conditions associated with recurrent exposure, such as Hymenoptera venom allergy, although the currently available evidence regarding the prognostic value of PAF-AH in this setting remains conflicting [37,38,39]. In parallel, improved characterization of PAF-related pathways may help identify selected patients who could potentially benefit from future adjunctive strategies targeting PAF synthesis, PAFR signaling, or PAF degradation [14,20,32,33,47]. These possibilities remain speculative at present and require prospective validation before translation into individualized clinical management.

At present, epinephrine remains the cornerstone of anaphylaxis treatment. Nevertheless, the accumulating experimental evidence supporting a role for PAF in severe reactions suggests that pharmacological modulation of the PAF pathway warrants further investigation. Future translational and clinical studies will be essential to determine whether PAF-directed therapies can contribute to improved prevention, risk reduction, or the treatment of anaphylaxis.

## 6. Conclusions

Anaphylaxis remains primarily a clinical diagnosis, and biomarkers currently play no role in its acute diagnosis and management. Nevertheless, the growing understanding of anaphylaxis endotypes has highlighted the potential value of molecular markers for improving diagnostic accuracy, risk stratification, and personalized management.

Among the mediators implicated in anaphylaxis, the PAF–PAF-AH axis has emerged as one of the most biologically plausible pathways associated with disease severity. Experimental and clinical evidence supports a role for excessive PAF activity in the amplification of vascular permeability, bronchospasm, hypotension, and cardiovascular dysfunction, while reduced PAF-AH activity appears to contribute to prolonged mediator bioavailability and more severe reactions.

Although current evidence does not support the routine clinical use of PAF or PAF-AH as diagnostic or prognostic biomarkers, these molecules remain valuable tools for understanding the mechanisms underlying severe anaphylaxis. Furthermore, the therapeutic potential of targeting the PAF pathway represents an area of active investigation and may provide novel opportunities for adjunctive intervention in high-risk patients.

Future studies should focus on assay standardization, validation in large multicenter cohorts, and the integration of PAF-related biomarkers within multimarker and systems biology approaches. Such efforts may ultimately contribute to improved patient stratification, a deeper understanding of anaphylaxis endotypes, and the development of biomarker-driven and targeted therapeutic strategies.

## Figures and Tables

**Table 1:** Key studies investigating the role of PAF and PAF-AH in anaphylaxis.

Study	Population/Model	Main Findings	Clinical Relevance
Fukuda et al., 2000 [32]	Murine models of anaphylactic shock	Administration of recombinant PAF-AH reduced hypotension and mortality during anaphylaxis.	First evidence that modulation of PAF activity may protect against severe anaphylaxis.
Vadas et al., 2008 [34]	Patients with food-, drug-, and venom-induced anaphylaxis	Circulating PAF levels increased with reaction severity, whereas PAF-AH activity showed an inverse correlation. Severe and fatal reactions were associated with the highest PAF levels and lowest PAF-AH activity.	Landmark study establishing the PAF–PAF-AH axis as a determinant of anaphylaxis severity.
Brown et al., 2013 [35]	402 anaphylaxis patients (mixed etiologies: drugs, foods, Hymenoptera stings, radiocontrast media, and idiopathic reactions)	Reduced PAF-AH activity was associated with hypotension, hypoxemia, and severe reactions.	Supported the role of impaired PAF degradation in severe anaphylaxis.
Pravettoni et al., 2014 [37]	169 patients with Hymenoptera venom allergy	Baseline PAF-AH activity was inversely associated with anaphylaxis severity. Patients with grades III–IV reactions had the lowest enzyme activity.	Suggested PAF-AH as a prognostic biomarker in venom-induced anaphylaxis.
Gill et al., 2015 [19]	Review of experimental and clinical evidence in human studies of anaphylaxis due to multiple triggers (primarily food, drugs, insect venom, and other IgE- and non-IgE-mediated causes)	Summarized the central role of PAF in vascular leakage, hypotension, and anaphylactic shock.	Consolidated mechanistic evidence linking PAF to severe anaphylaxis.
Piwowarek et al., 2021 [38]	89 patients with Hymenoptera venom allergy and controls	Plasma PAF-AH activity was significantly lower in patients with a history of anaphylaxis than in the controls.	Supported the potential utility of PAF-AH in identifying high-risk individuals.
Bilò et al. 2022 [39]	103 selected patients with Hymenoptera venom allergy, compared with real-world patients, healthy subjects and patients with allergic rhinitis or asthma.	Lower PAF-AH levels in HVA patients, but no association with reaction severity.	Suggests that reduced PAF-AH is a marker of Hymenoptera venom allergy but it has limited value for predicting severe venom-induced anaphylaxis.
Upton et al., 2022 [36]	46 pediatric patients with acute anaphylaxis (mixed etiologies: food, drugs, insect stings, and idiopathic).	Low PAF-AH activity was strongly associated with life-threatening reactions and intensive care admission.	Demonstrated the potential value of PAF-AH as a severity biomarker in children.
Suzuki et al., 2025 [33]	Experimental model of cutaneous anaphylaxis	Identified mast cell LPLAT9 as a key enzyme driving PAF synthesis during IgE-mediated allergic responses.	Provided novel mechanistic insights into PAF generation during anaphylaxis.

PAF, platelet activating factor; PAF-AH, platelet activating factor-acetylhydrolase.

**Table 2:** Some biomarkers investigated in anaphylaxis.

Biomarker	Advantages	Limitations	Clinical Use
Tryptase	Widely available	Often normal in food anaphylaxis	Routine
Histamine	Direct mediator	Short half-life	Limited
PAF	Strong correlation with severity	Difficult to measure, very short half-life	Research
PAF-AH	More stable than PAF	Conflicting evidence	Research
CCL2	Emerging marker	Limited validation	Research
Chymase	Potential adjunct marker	Not routinely available	Research

## Data Availability

No new data were created or analyzed in this study. Data sharing is not applicable to this article.
